# Supplementary material for: Transcriptional Memory-Like Imprints and Enhanced Functional Activity in γδ T Cells Following Resolution of Malaria Infection
Source: Front Immunol. 2020 Oct 14;11:582358. doi: 10.3389/fimmu.2020.582358 (PMC7591758; doi:10.3389/fimmu.2020.582358)
Supplement: Supplementary file 1 [file DataSheet_1.pdf]

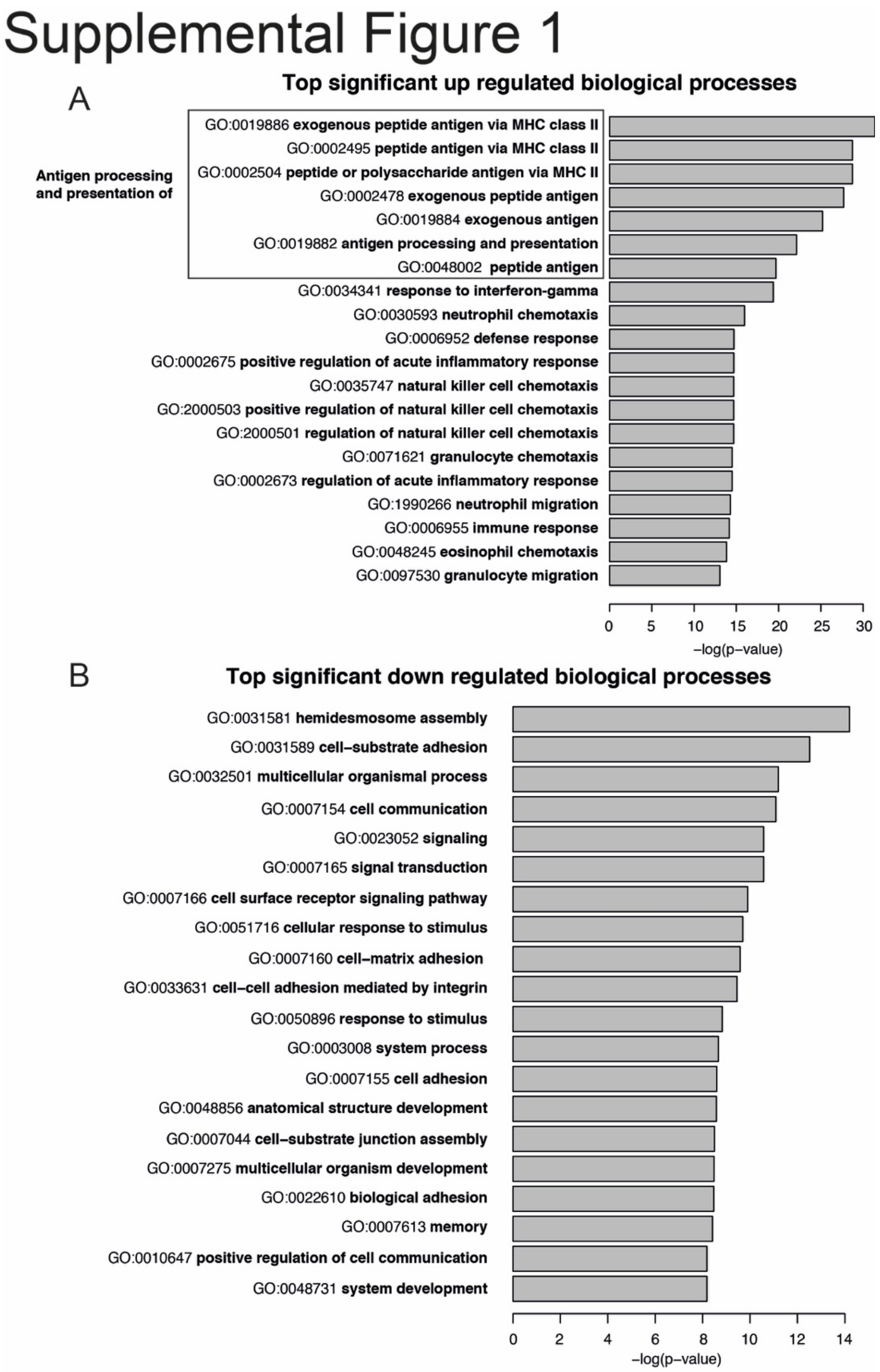

Supplemental Figure 1. Pathway analysis of differentially expressed genes. Gene Ontology (GO) terms for the differentially expressed genes were identified and the top 20 significantly A) upregulated and B) down regulated biological processes are presented.

Supplemental Figure 2

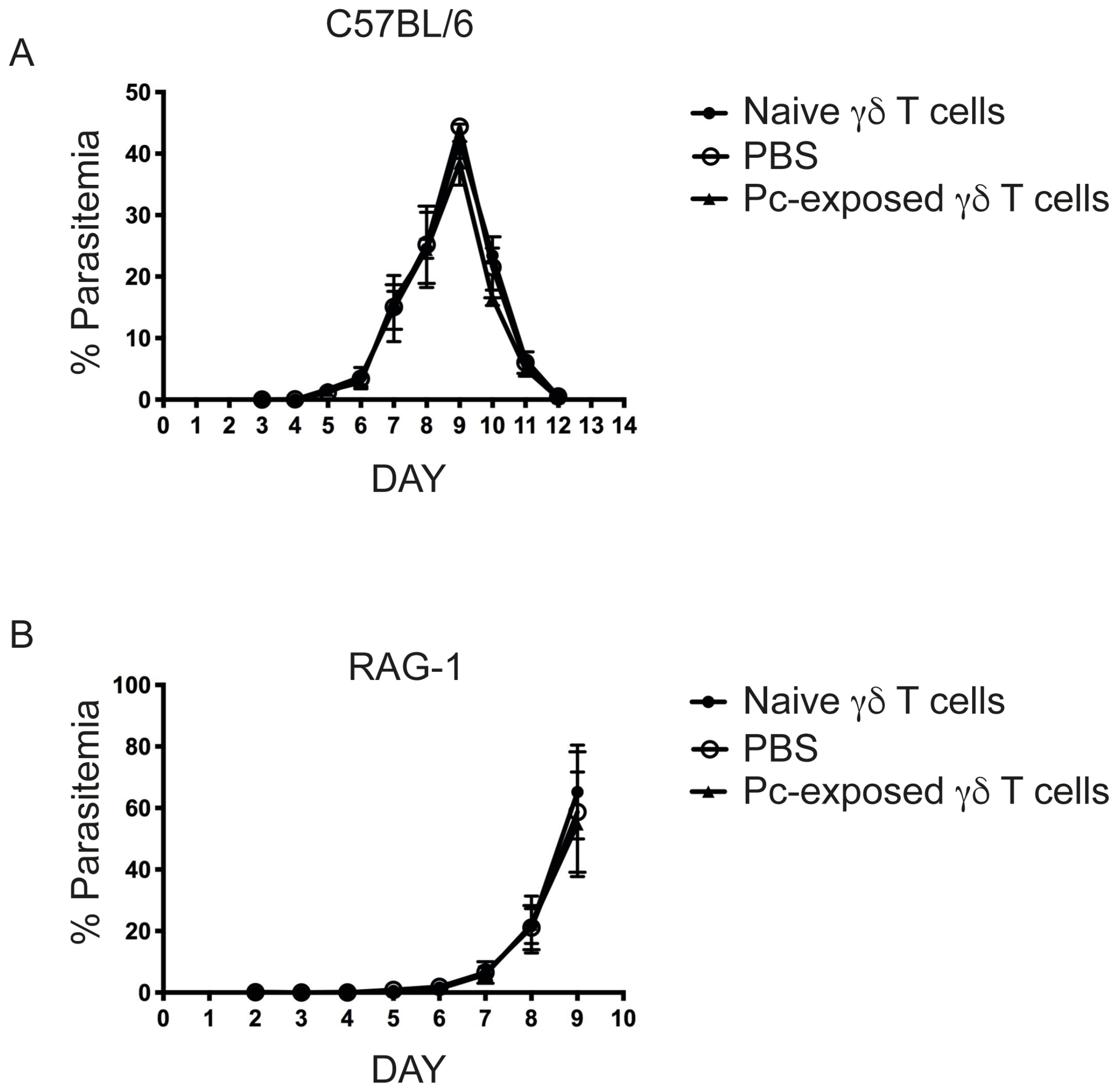

Supplemental Figure 2. Parasitemia following adoptive transfer of *P. chabaudi*-exposed γδ T cells. γδ T cells were isolated from spleens of naïve mice (filled circle) or *P. chabaudi*-exposed mice (filled triangle). These cells were then adoptively transferred into A) C57BL/6 mice (Naïve γδ T cells n=4, Pc-exposed γδ T cells n=4, PBS n=3) or B) RAG-1 mice ((Naïve γδ T cells n=7, Pc-exposed γδ T cells n=6, PBS n=8) 1 day post infection of recipient mice. Control mice received no cells, but were injected with PBS (open circles) 1 day post infection. Parasitemia was determined daily by thin blood smears. Statistical analysis was performe using Multiple t-tests, Holm-Sidak method.

Supplemental Table 1. Differentially expressed genes in resting EM γδ T cells for *P. chabaudi* exposed mice over naïve mice relative to a fold change threshold of 1.5.

GeneID	Symbol	logFC	P.Value	adj.P.Val
16640	Klra9	3.39044649	1.42E-08	0.00018394
20302	Ccl3	2.61819118	3.59E-08	0.00023202
140919	Slc17a6	8.58584287	1.29E-07	0.00055684
20303	Ccl4	2.92039006	2.31E-07	0.00074802
17181	Matn2	-3.0656979	7.88E-07	0.00175484
20668	Sox13	-2.9775554	8.14E-07	0.00175484
14961	H2-Ab1	3.34151569	1.29E-06	0.00209603
14969	H2-Eb1	3.06629838	1.47E-06	0.00209603
16639	Klra8	3.39858743	1.58E-06	0.00209603
244233	Cd163l1	-3.6734629	1.63E-06	0.00209603
192897	Itgb4	-4.2356539	1.78E-06	0.00209603
104001	Rtn1	6.32010768	2.01E-06	0.00210703
320712	Abi3bp	-2.687706	2.24E-06	0.00210703
14960	H2-Aa	3.74438646	2.28E-06	0.00210703
67971	Tppp3	-3.3576541	3.87E-06	0.00333899
67784	Plxnd1	-3.6726208	5.56E-06	0.00445659
76933	Ifi27l2a	1.9771099	5.86E-06	0.00445659
16149	Cd74	2.93113707	7.00E-06	0.00484936
16407	Itgae	-1.9508531	7.15E-06	0.00484936
16525	Kcnk1	-2.2320014	7.98E-06	0.00484936
108105	B3gnt5	-2.1318702	8.00E-06	0.00484936
75124	Nxnl2	-3.9675443	8.25E-06	0.00484936
64074	Smoc2	3.22005352	9.93E-06	0.00558654
320940	Atp11c	-3.0596644	1.18E-05	0.0063155
14127	Fcer1g	2.28114455	1.22E-05	0.0063155
22700	Zfp40	-2.8183597	1.34E-05	0.00667617
16628	Klra10	3.17360354	1.52E-05	0.00728868
56620	Clec4n	5.47291964	1.61E-05	0.00737075
16728	L1cam	-2.6150969	1.70E-05	0.00737075
16177	Il1r1	-3.0255573	1.71E-05	0.00737075
94180	Acsbg1	-2.1520114	1.78E-05	0.00742548
434179	Zfp975	-2.7566189	1.93E-05	0.00781934
12143	Blk	-2.0813922	2.03E-05	0.00786837
109689	Arrb1	-2.4470744	2.12E-05	0.00786837
105298	Epdr1	2.29149016	2.13E-05	0.00786837
66857	Plbd1	3.1880755	2.23E-05	0.00800138
27403	Abca7	-2.2961181	2.44E-05	0.00853689
19225	Ptgs2	5.48449503	2.92E-05	0.00951065
240034	Zfp760	-2.6778093	2.94E-05	0.00951065
20304	Ccl5	1.80542457	2.94E-05	0.00951065
12959	Cryba4	-3.1216169	3.33E-05	0.0104986
217169	Tns4	-3.4001073	3.66E-05	0.0111969
320832	Sirpb1a	4.11075448	3.72E-05	0.0111969
15000	H2-DMb2	3.4752987	4.52E-05	0.01299018
217166	Nr1d1	-1.8385267	4.55E-05	0.01299018
23833	Cd52	1.54680848	4.62E-05	0.01299018
408068	Zfp738	-2.231938	5.04E-05	0.01382819
20441	St3gal3	-2.4250712	5.16E-05	0.01382819
16541	Napsa	1.82045567	5.24E-05	0.01382819
73218	Sppl2b	-1.9845924	5.47E-05	0.01415938
22177	Tyrobp	2.03778719	5.85E-05	0.01470045
69623	Zfp33b	-2.2383166	6.00E-05	0.01470045
327957	Scimp	2.36750583	6.24E-05	0.01470045
231507	Plac8	2.10617985	6.26E-05	0.01470045
27375	Tjp3	-1.9661036	6.27E-05	0.01470045
18810	Plec	-2.0152577	6.45E-05	0.01470045
13924	Ptprv	-2.1715927	6.55E-05	0.01470045
52673	D13Ertd608e	6.07348703	6.59E-05	0.01470045
105844	Card10	-2.6295919	6.85E-05	0.0147293
16449	Jag1	-2.2705059	6.91E-05	0.0147293
26888	Clec4a2	7.36981245	6.95E-05	0.0147293
11828	Aqp3	-1.9149296	7.31E-05	0.0150935
72310	Nkg7	1.49537211	7.35E-05	0.0150935
227737	Fam129b	-2.4930972	7.68E-05	0.01551876
16419	Itgb5	-2.518283	8.26E-05	0.01635881
240047	Mmp25	-2.021048	8.35E-05	0.01635881
68836	Mrpl52	1.71245827	8.63E-05	0.0166606
677296	Fcrl6	2.52556764	9.08E-05	0.01714184
76089	Rapgef2	-2.4179422	9.26E-05	0.01714184
14999	H2-DMb1	2.6663498	9.30E-05	0.01714184
235627	Nbeal2	-2.2397252	9.41E-05	0.01714184
13590	Lefty1	3.91201824	9.83E-05	0.01766426
15978	Ifng	1.54412634	0.00010009	0.01773421
234839	Piezo1	-1.8827456	0.00010328	0.01788065
11501	Adam8	-2.024776	0.00010561	0.01788065
19216	Ptger1	-2.4559706	0.00010627	0.01788065
17067	Ly6c1	1.77670531	0.00010645	0.01788065
74691	Tdrd9	-4.894266	0.00011056	0.01806239
64009	Syne1	-2.2244327	0.00011247	0.01806239
57294	Rps27	1.81403551	0.00011273	0.01806239
545389	Cep170	-2.0176272	0.00011312	0.01806239
235626	Setd2	-2.0350638	0.00011845	0.01868366
13733	Adgre1	3.28728561	0.00012078	0.01881755
69698	Slc52a3	-2.3118547	0.00012221	0.01881755
69781	1600010M07	6.00329433	0.00013267	0.02018718
100041546	Ly6c2	1.52389971	0.00013728	0.02064608
224697	Adamts10	-1.7600819	0.00014343	0.02130377
269254	Setx	-1.9703498	0.00014495	0.02130377
74194	Rnd3	4.81909751	0.00014927	0.02169261
12045	Bcl2a1b	1.56732395	0.00015629	0.0220893
16905	Lmna	-2.1187115	0.00015995	0.0220893
217082	Hlf	-2.6080009	0.00016201	0.0220893
12047	Bcl2a1d	1.58363573	0.00016267	0.0220893
546643	I830127L07R	2.64517731	0.00016386	0.0220893
231380	Uba6	-1.9325607	0.00016425	0.0220893
20379	Sfrp4	5.47280312	0.00016729	0.0220893
213498	Arhgef11	-2.4534256	0.0001683	0.0220893
19401	Rara	-1.6487025	0.00016845	0.0220893
11958	Atp5k	1.71041274	0.00017328	0.0220893
547023	Gm6014	1.59037474	0.00017408	0.0220893
100038467	A630072L19I	-3.1879796	0.00017412	0.0220893
104759	Pld4	2.23339953	0.0001742	0.0220893
20335	Sec61g	1.71316333	0.00018335	0.02285935
66141	Ifitm3	3.1891748	0.00018393	0.02285935
16963	Xcl1	1.67046898	0.00018558	0.02285935
333789	N4bp2	-2.1499791	0.00020016	0.0244229
93736	Aff4	-1.6704461	0.00020374	0.02462745
18636	Cfp	2.5068555	0.00021903	0.0261136
19724	Rfx1	-1.7988245	0.00022007	0.0261136
99031	Osbpl6	-5.4399794	0.00022644	0.02650221
242474	Tmem245	-1.9398074	0.00022926	0.02650221
105203	Fam208b	-2.1364652	0.00022949	0.02650221
14419	Gal	5.23497664	0.0002367	0.02703276
27278	Clnk	-3.1480376	0.00023855	0.02703276
106064	AW549877	-1.4509389	0.00024242	0.02703276
58800	Trpm7	-1.6551586	0.00024546	0.02703276
100114901	Trdv4	-3.1632699	0.0002466	0.02703276
546336	Prrg1	-3.7274869	0.00024663	0.02703276
100042480	Nhsl2	-2.3384695	0.00024908	0.02707175
70882	Armc3	2.09645514	0.00025411	0.02738847
212281	Zfp729a	-1.8037739	0.00026404	0.02822362
11305	Abca2	-2.1059443	0.00026962	0.02849805
433771	Minos1	1.46099902	0.00027444	0.02849805
18798	Plcb4	-1.8991147	0.00027573	0.02849805
668548	Gm9234	1.5886041	0.00027762	0.02849805
19942	Rpl27	1.49528932	0.000279	0.02849805
13848	Ephb6	-2.1407699	0.0002806	0.02849805
623286	Gm6415	1.73980233	0.00028276	0.02849805
64380	Ms4a4c	1.56184576	0.0002846	0.02849805
18799	Plcd1	-2.9029124	0.00028877	0.02849805
232157	Mob1a	-1.8235198	0.00029138	0.02849805
100310809	Gm10509	-1.9455024	0.00029467	0.02849805
85030	Tnfrsf25	-1.5834613	0.00029471	0.02849805
20874	Slk	-1.6840805	0.00029525	0.02849805
80876	Ifitm2	3.33832857	0.00030793	0.02936592
70747	Tspan2	-3.1933786	0.00031025	0.02936592
217344	Rhbdf2	-1.9191638	0.00031105	0.02936592
17970	Ncf2	2.34277338	0.00032008	0.02985542
74206	Sipa1l3	-2.870379	0.00032085	0.02985542
57295	Icmt	-2.7002589	0.00032518	0.03004208
103012	Firre	-2.885473	0.00033104	0.03036672
235682	Zfp445	-1.6368334	0.00033843	0.03065132
664903	Rps15a-ps4	1.52584779	0.00033889	0.03065132
71679	Atp5h	1.46354865	0.00034347	0.03085009
595139	E030024N20	1.9564233	0.00035041	0.03105678
54127	Rps28	1.63489106	0.00035057	0.03105678
442834	D830031N03	-5.6264778	0.00035918	0.03160264
232286	Tmf1	-1.841913	0.00037438	0.03271751
209590	Il23r	-1.5742096	0.00038148	0.03311473
14255	Flt3	3.93307654	0.00039181	0.03343625
68052	Rps13	1.52797482	0.00039619	0.03343625
76486	Ly6k	2.43981225	0.00039855	0.03343625
56318	Acpp	-1.7027226	0.00039856	0.03343625
233115	Dpy19l3	-1.8132615	0.00039868	0.03343625
244234	5830411N06	-1.8919018	0.00040359	0.03343625
14710	Gngt2	1.59211673	0.00040515	0.03343625
226691	Ifi207	4.14276731	0.00040587	0.03343625
14934	Gypa	4.47460481	0.00042532	0.03481664
27762	Vwa7	-3.8726915	0.00044147	0.03564666
100217422	Snord13	2.4390167	0.00044415	0.03564666
70081	Zfp995	-2.0314871	0.0004466	0.03564666
11798	Xiap	-1.8115982	0.0004494	0.03564666
240753	Plekha6	-3.2662489	0.00044954	0.03564666
66475	Rps23	1.53622924	0.00045199	0.03564666
218850	Fam208a	-1.7662213	0.00047048	0.03688001
100043695	Gm15483	1.65788997	0.00048099	0.03714473
29816	Hip1r	-1.7443726	0.00048107	0.03714473
268373	Ppia	1.49147232	0.00048247	0.03714473
27356	Insl6	1.77847073	0.00049245	0.03768816
226695	Ifi205	1.60727328	0.00050021	0.03784678
24074	Taf7	1.42555474	0.00050037	0.03784678
268449	Rpl23a	1.57644911	0.00051487	0.03871673
72672	Zfp518a	-1.5869731	0.00051826	0.03874669
14125	Fcer1a	5.87666897	0.00054134	0.04023936
100043882	Gm14277	1.55978207	0.00054489	0.0402722
67671	Rpl38	1.87136115	0.00054916	0.04035677
211673	Arfgef1	-1.4996966	0.00055826	0.04079382
16440	Itpr3	-1.7847781	0.0005751	0.04178855
100043296	Gm4342	1.47870869	0.00057988	0.0419007
245671	Klf8	-4.9847596	0.00058537	0.04206218
54153	Rasa4	-1.9113718	0.00060161	0.04283607
77700	9130208D14	-2.4595416	0.00060277	0.04283607
73656	Ms4a6c	1.80779298	0.00060755	0.04294023
240038	Zfp994	-1.7955281	0.00061668	0.04334875
381605	Tbc1d2	-3.9868935	0.00062098	0.04341456
100042335	Rps15a-ps5	1.50810763	0.00062536	0.04348578
230787	Themis2	2.91912847	0.00063685	0.04378543
14130	Fcgr2b	4.14352871	0.00064223	0.04378543
633188	Gm20762	1.53664557	0.00064238	0.04378543
100124677	Trbv13-3	1.88412132	0.00064321	0.04378543
226519	Lamc1	-2.9979216	0.0006607	0.04451856
73945	Otud4	-1.6303022	0.00066086	0.04451856
100042173	Rps15a-ps6	1.64503684	0.00066518	0.04457726
14104	Fasn	-2.1972034	0.0006725	0.04483534
667090	Gm8451	1.6598696	0.0006883	0.04547373
16402	Itga5	-1.8821888	0.00068999	0.04547373
20602	Ncor2	-1.9287641	0.00069262	0.04547373
242202	Pde5a	-1.6322369	0.00069731	0.04555069
11690	Alox5ap	3.92836672	0.00070132	0.04558242
213121	Ankrd35	-2.7322028	0.00072014	0.0465715
319565	Syne2	-1.6112692	0.00073176	0.04683652
100416706	Zfp729b	-1.5515733	0.00073552	0.04683652
19861	Rnu3b4	1.65181887	0.00073606	0.04683652
19941	Rpl26	1.45725058	0.00073872	0.04683652
268670	Zfp759	-3.0108621	0.00078001	0.04914534
11941	Atp2b2	3.64003229	0.0007833	0.04914534
16909	Lmo2	2.95502143	0.00078654	0.04914534
57890	Il17re	-1.4806967	0.00079281	0.04929907
74002	Psd2	-3.1439883	0.00079855	0.04941853
100134990	Selenok-ps1	1.71535575	0.00080446	0.04954681
19850	Rnu3a	2.22061571	0.00081795	0.0499763
226641	Atf6	-1.767882	0.00082072	0.0499763
100039988	Gm11826	1.85151395	0.00082302	0.0499763
